# Addressing mental health challenges in clinical practice: a qualitative study to investigate perspectives of international mental health experts

**DOI:** 10.1007/s00406-025-02064-2

**Published:** 2025-10-23

**Authors:** Melissa G. Halil, Anissa Abi-Dargham, John H. Krystal, David M. Clark, Adrian P. Mundt, Helen Herrman, Solomon Rataemane, Fritzi Weitzenegger, Isabel Dziobek, Andreas Heinz, Irina Baskow

**Affiliations:** 1 Department of Psychiatry and Psychotherapy, Charité Universitätsmedizin Berlin, Charité Campus Mitte (CCM), Charitéplatz 1, 10117 Berlin, Germany; 2 German Center for Mental Health (DZPG), Partner Site Berlin-Potsdam, Berlin, Germany; 3 Department of Psychiatry and Behavioral Health, Renaissance School of Medicine, Stony Brook University, Stony Brook, NY USA; 4 Yale University School of Medicine, New Haven, CT USA; 5 Veterans Affairs Connecticut Healthcare System, West Haven, CT USA; 6 Oxford Centre for Anxiety Disorders and Trauma (OxCADAT), Department of Experimental Psychology, The Old Rectory, University of Oxford, Paradise Square, Oxford, OX1 1TW UK; 7 Centro de Investigación Biomédica, Medical Faculty, Universidad Diego Portales, Santiago, Chile; 8 Department of Psychiatry and Mental Health, Hospital Clínico Universidad de Chile, Santiago, Chile; 9 Orygen and Centre for Youth Mental Health, The University of Melbourne, Parkville, VIC Australia; 10 Kindred Collaborative, Cairns, QLD Australia; 11 Department of Psychiatry, Sefako Makgatho Health Sciences University, Ga-Rankuwa, South Africa; 12 Department of Psychiatry and Psychotherapy, Charité Universitätsmedizin Berlin, Campus Benjamin Franklin (CBF), Berlin, Germany; 13 Klinische Psychologie Sozialer Interaktion, Institut für Psychologie, Humboldt- Universität zu Berlin, Unter den Linden 6, 10099 Berlin, Deutschland; 14 Department of Psychiatry and Psychotherapy, Tübingen Center for Mental Health (TüCMH), Medical School and University Hospital, Eberhard Karls University of Tübingen, Tübingen, Germany; 15 German Center for Mental Health (DZPG), Partner Site Tübingen, Tübingen, Germany

**Keywords:** German center for mental health (DZPG), Mental health research, Translational psychiatry, Focus group study, Social determinants of mental health, Precision psychiatry

## Abstract

**Introduction:**

Mental illnesses are not only associated with the highest level of subjective suffering compared to other chronic diseases but at the same time with a significantly increased morbidity. Especially in industrialized countries, a dramatically increasing proportion of sick leave and early retirement due to mental illness diagnoses is observed, pointing to increased social stress factors and a close association with societal changes and emphasizing challenges to the health care system. The following qualitative study was conducted as part of the establishment of the German Center for Mental Health, which unites excellence in psychiatric, psychological and neuroscientific research in Germany to facilitate translation of basic into clinical research and general health care.

**Methods:**

A 120-minute, guided, virtual focus group discussion was conducted with international experts in the field of mental health research from Chile, the USA, the UK, South Africa, and Australia to represent views from different continents. The participants discussed international trends, unrecognized needs and gaps in clinical practice and recognized many opportunities and challenges in the research of the different topics of the German Center for Mental Health. Furthermore, the focus areas of the German Center were presented, and participants were asked about the opportunities and challenges in researching these topics. The evaluation method was based on qualitative content analysis according to Mayring. The focus group discussion was transcribed and coded with MAXQDA 2022 software. The reliability of the coding was checked by using the intercoder reliability and Cohens Kappa.

**Results:**

The experts emphasized the high mortality due to comorbidity of somatic and mental disorders including addiction and called for a mechanism-based approach to promote individualized treatment. Furthermore, the impact of poverty, social exclusion, stigma and discrimination was addressed as a key modifiable environmental risk factor. The experts highlighted digital tools, computational models and AI based approaches for precision psychiatry. To systematically evaluate real-world outcomes, the experts emphasized the need for interdisciplinary research including behavioral, computational, social, and neurobiological scientists and experts by experience.

**Discussion:**

This study highlights the need for interdisciplinary and participatory approaches in mental health research. Key challenges include the high comorbidity of mental and physical disorders, the impact of social determinants, and gaps in translating research into practice. Experts emphasized the importance of digital tools, computational psychiatry, and community-based interventions to enhance prevention and treatment. The German Center for Mental Health (DZPG) is well-positioned to address these challenges by integrating diverse expertise and fostering innovation in mental health care.

## Introduction

Mental disorders are among the most common health conditions, with a high prevalence, often early onset, and chronic courses that significantly impact individuals and society. In Germany, approximately one in four people is affected [1], and despite extensive research efforts, the overall burden of mental illness has not decreased in recent decades. Contributing factors include limited understanding of etiology, insufficient early detection, and a lack of targeted preventive and therapeutic strategies. Additionally, socially disadvantaged and marginalized groups, such as unemployed individuals and people with migration backgrounds, are particularly affected by mental health conditions and face additional barriers to adequate care [2]. To address these challenges, the German Federal Government established the German Center for Mental Health (DZPG) as part of the German Centers for Health Research [3]. The DZPG’s mission is to optimize research conditions, accelerate the translation of findings into clinical application, and significantly improve mental health care through interdisciplinary collaboration, involving medical professionals, psychologists, social scientists, and the perspectives of individuals with lived experience [4].

The DZPG operates across six sites: Berlin-Potsdam, Bochum-Marburg, Halle-Jena-Magdeburg, Mannheim-Heidelberg-Ulm, Munich-Augsburg, and Tübingen. These locations were selected through a competitive process in 2021 [5] and have since developed a joint research concept aimed at fostering scientific collaboration, ensuring optimal conditions for innovative mental health research, and facilitating the rapid implementation of findings into practice. The DZPG collaborates with 42 university hospital departments and 139 affiliated teaching hospitals, collectively treating over 48,500 inpatients and 224,000 outpatients annually. This extensive network enables the development of harmonized infrastructure, standardized recruitment and data management strategies, and interoperable platforms that integrate research, education, and clinical care across all six sites [6, 7]. A particular focus is placed on addressing mental health inequalities, particularly in urban and rural settings, and ensuring that vulnerable populations receive targeted support.

**Fig. 1:**
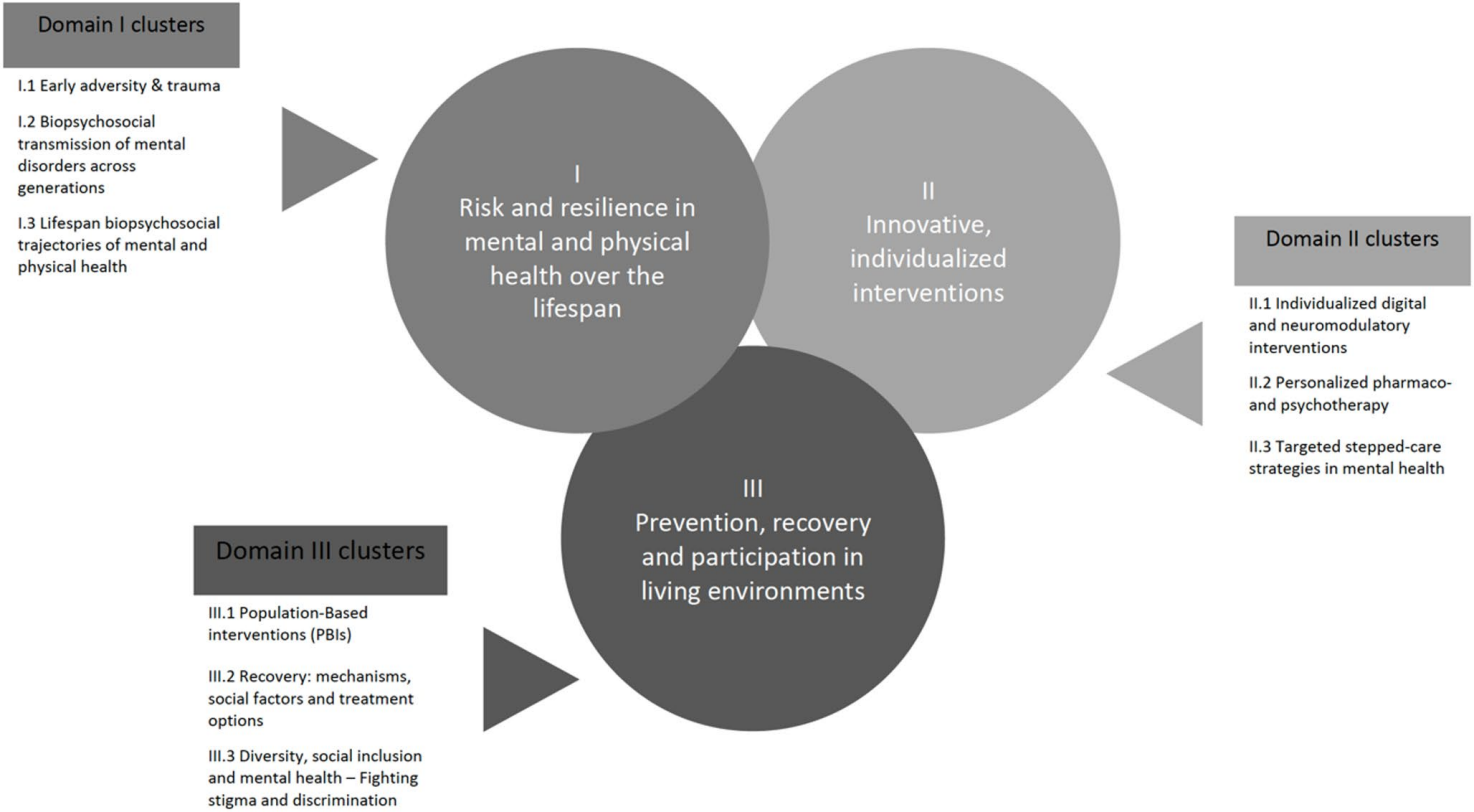
Domains and Clusters of the German Center for Mental Health. Domains can be seen in the circles (I– III) and three cluster for every domain can be found in the boxes next to the circles

The research framework of the DZPG is structured into three core domains, each encompassing specific research priorities. The first domain, Risk and Resilience in Mental and Physical Health Across the Lifespan, investigates biopsychosocial risk and protective factors through deep phenotyping, longitudinal studies, and advanced modeling techniques to better understand individual disease trajectories [8]. The second domain, Innovative and Individualized Interventions, aims to develop novel digital, pharmacological, and psychotherapeutic approaches, including neuromodulatory interventions, to optimize treatment effectiveness [9, 10]. The integration of neuroscience-based approaches in psychiatric research has been proposed to enhance biological treatment frameworks [11, 12]. The third domain, Prevention, Recovery, and Participation in Living Environments, focuses on the societal and environmental determinants of mental health, aiming to reduce stigma and enhance participation and recovery within communities [13]. Each domain consists of three clusters that further specify key areas of translational research (Fig. 1).

Additionally, three flagship projects have been identified as areas of particular translational relevance. Urban Mental Health addresses mental health challenges in metropolitan environments, where exposure to social stressors, discrimination, and economic hardship increases the risk of mental illness [14]. Early Recognition, Intervention, and Prevention focuses on early disease detection and intervention strategies, particularly in children and adolescents, as three-quarters of mental disorders develop before the age of 25 [15, 16]. Enhanced Psychotherapy explores the integration of psychotherapeutic and biological interventions, combining psychotherapy with neuromodulation, pharmacology, and digital tools to enhance treatment outcomes [17].

A key element of the DZPG is its strong commitment to participatory research [18, 19]. From the outset, individuals with lived experience, patients, and family members have been systematically involved in decision-making processes, ensuring that research is relevant, feasible, and responsive to real-world needs. A Trialogic Center Council, established early in the process, integrates patient and family perspectives into all research activities. Furthermore, local advisory councils at each site provide additional opportunities for participatory engagement, making the DZPG a pioneer in systematically embedding Patient and Public Involvement (PPI) in mental health research [20].

Looking ahead, the DZPG aims to further expand its research activities by strengthening its translational infrastructure, enhancing data harmonization across sites, and increasing its focus on underrepresented populations. The next steps include refining the implementation of adaptive platform trials, strengthening interdisciplinary collaborations, and securing long-term funding to sustain its research objectives. Through interdisciplinary and participatory approaches, the DZPG seeks to drive innovation in mental health prevention, treatment, and recovery at both the individual and societal levels. To evaluate the development and achievements of this new center, an international advisory board was established, and the members were contacted for a first focus group on key challenges and chances in mental health research and care, the results of which are presented below.

## Aims of the focus group and key discussion points

To align the new center with international research trends, current issues in mental health, and gaps in translating findings from basic to clinical research, we invited six international experts on mental health to participate in a focus group:

- Neuroimaging and the neurobiological mechanisms of schizophrenia, with a particular focus on dopamine dysregulation (Stony Brook University, United States).
- Innovative pharmacological and neurobiological approaches to addiction and mood disorders, including cutting-edge research on ketamine’s antidepressant effects (Yale University School of Medicine, United States).
- The development, evaluation, and international dissemination of cognitive behavioral therapy for anxiety disorders, shaping contemporary psychological treatment standards (University of Oxford, United Kingdom).
- Global mental health and psychiatric epidemiology, with a special focus on mental health care systems and policy development in Latin America (Universidad Diego Portales, Chile).
- Public mental health, youth mental health interventions, and the integration of psychiatric services into community-based care models (University of Melbourne, Australia).
- Mental health policy and addiction psychiatry, particularly in the context of healthcare infrastructure and psychiatric service delivery in Africa (Sefako Makgatho Health Sciences University, South Africa).

The participants were asked to (1) analyze international research trends in mental.

health, (2) identify unmet needs that includes the needs of patients, family members, and professionals, and (3) examine existing gaps in clinical practice and research. The group of experts evaluated how the proposed research areas and methodological platforms should be modified to enhance excellence and international competitiveness. Guided qualitative focus groups were conducted with three expert panels to gather these insights [21]. This paper focuses on the international expert panel.

Given the high prevalence and burden of mental illnesses in Germany, as well as the limited effectiveness of current pharmacological and non-pharmacological treatments, the study addressed four key research questions:

- Q1: What research trends are currently taking place in the field of mental health in other countries?
- Q2: Where do you see gaps and needs in clinical practice?
- Q3: How can gaps in the translation of research findings and guidelines into clinical practice be addressed?
- Q4: The DZPG is organized into three collaborative research areas that focus on (1) the high somatic comorbidity and increased mortality of people with mental disorders across the lifespan, (2) the impact of poverty, social exclusion, stigma, and discrimination, and (3) how knowledge from behavioral, computational, social, and neurobiological sciences can be used to facilitate better treatments and optimize outcomes. Where do you see opportunities and challenges in researching these topics?

By addressing these questions, the focus groups aimed to provide valuable guidance for the future direction of the German Center for Mental Health. The insights gained serve as a foundation for optimizing research structures and ensuring that the Center aligns with both national and international priorities in mental health research.

## Methods

### Focus groups

A focus group is a moderated discussion with multiple participants based on a guideline with open-ended questions [22]. This method has been widely used in various research fields, including health and social sciences, due to its ability to generate insights through group interaction [23–25]. Virtual focus groups, conducted via a team collaboration application, provided an efficient and cost-effective means of data collection, allowing participants to express their perspectives in a structured discussion format [26, 27]. All sessions were recorded for subsequent transcription and analysis. The focus group was conducted using a dual-moderation approach, ensuring structured discussion and documentation.

### Ethics and consent

The study was conducted under the ethics application “Recognizing Diversity for Optimized Treatment and Outcomes”, approved in 2021. Participants provided written informed consent, which included an explanation of the study’s purpose, data protection measures, and participant rights. Personal identifiers such as name, title, and affiliation were pseudonymized using a five-digit alphanumeric code. All data were securely stored, and recordings were deleted upon project completion in compliance with data protection regulations (BlnDSG; EU-GDPR).

### Participants

The focus group participants (*n* = 6; 2 female and 4 male experts; average age = 60 years) were nominated by the Berlin-Potsdam site board to join the International Scientific Advisory Board of the German Center for Mental Health. The selection was based on scientific standing, including publication record and h-index, and aimed to ensure broad international representation by initially inviting one expert per continent. Due to scheduling constraints, not all invitees were able to participate in the focus group discussion. The final panel included internationally recognized experts from North America, South America, Europe, Africa, and Australia. Their diverse backgrounds in psychiatry, neuroscience, addiction research, and social determinants of mental health ensured a broad range of perspectives relevant to the German Center for Mental Health’s research agenda.

### Discussion guidelines

A guideline was sent to participants two weeks prior to the focus group, providing information on the purpose of the discussion, technical instructions for Microsoft Teams, and key discussion topics. Ground rules for open and respectful dialogue were also included.

### Study design

Data were analyzed using a structured qualitative content analysis approach [28]. A theory-guided coding system was developed and iteratively refined through data-driven modifications. Communicative validation was applied by presenting preliminary findings to participants, ensuring alignment with expert perspectives. To enhance methodological rigor, peer debriefing was conducted at multiple stages of analysis [29, 30], Transcription was conducted using MAXQDA2022 (Analytics Pro, version 2022.2.1) software. Recordings were transcribed verbatim by a research assistant and verified by two additional team members for accuracy. Unclear audio segments were reviewed with native speakers or analyzed in slow-motion playback.

### Coding framework

The coding process was divided into Basic Coding and Fine Coding [31]. Basic Coding was conducted using the MAXQDA “word cloud tool,” extracting the most frequently mentioned terms in the focus group discussion. Only verbs, nouns, and adjectives were considered, while adverbs, articles, pronouns, prepositions, connectors, particles, and interjections were excluded. A cut-off frequency of ≥ 10 occurrences was applied.

To ensure comprehensive coverage, additional key terms from the application text for the concept development phase—such as Early Adversity, Computational Psychiatry, and Stigmatization—were incorporated. These terms were mapped onto Fine Coding, aligning them with the three research domains of the German Center for Mental Health: Risk and Resilience, Innovative Individualized Treatments, and Prevention, Recovery, and Participation. To enhance completeness, two additional categories -Translation and Structures and Procedures - were included.

In Fine Coding, terms related to Risk and Resilience included Substance Use, Biomarkers, Early Adversity, Trauma, Urban Mental Health, and Biopsychosocial Transmission Across Generations. These were categorized as contributing to understanding the causes, trajectories, and resilience mechanisms related to mental disorders. The Innovative Individualized Treatments category encompassed terms such as Interventions, Pharmacotherapy, Psychotherapy, Computational Psychiatry, Biomarkers, Neuroscience, and Neurostimulation, reflecting efforts to optimize patient benefits through precise, scalable, and mechanistically driven therapies. The Prevention, Recovery, and Participation category included Population-Based Interventions, Cohesion in Mental Health Care, Stigmatization, Diversity, and Social Inclusion/Exclusion, emphasizing the integration of mental health interventions into real-world settings. The Translation category addressed how improved predictive modeling could be embedded into intervention studies and translated into healthcare services. Lastly, Structures and Procedures covered organizational aspects, including Funding, Infrastructure, Governance, and Patient and Public Involvement (PPI).

### Intercoder reliability

Intercoder reliability is an essential instrument in qualitative content analysis, ensuring that multiple coders independently apply the category system consistently [28]. It allows verification of whether the categories are clearly formulated and selective [29]. After coding, the results are compared to assess agreement. Achieving high agreement can be challenging, especially in the first coding pass, often leading to category system revisions. Despite ongoing debates about its significance [32], intercoder reliability remains a fundamental quality criterion in content analysis and contributes to refining the coding framework.

For this study, intercoder reliability was calculated using Cohen’s Kappa, which corrects for chance agreement [33]. According to [34] and Landis & Koch [35], kappa values between 0.61 and 0.80 are considered “substantial,” and values above 0.81 “almost perfect.” A second research assistant coded the material using MAXQDA, with all irrelevant sequences (e.g., small talk and introduction rounds) blacked out. The assistant received all basic codes and fine code definitions for orientation. The only instruction was that coding should be applied to full sentences or independent sentence segments rather than isolated words. The agreement rates varied across different categories. The highest agreement was observed for Prevention, Recovery, and Participation, with 40 consistent codings and 4 inconsistencies, resulting in an agreement rate of 90.91%. Innovative and Individualized Treatments had 66 consistent codings and 19 inconsistencies, leading to an agreement of 77.65%. Structures and Procedures of the GCMH showed 10 consistent codings and 3 inconsistencies (76.92% agreement), while Risk and Resilience reached 74.07% agreement (30 consistent, 14 inconsistent). The lowest agreement was observed in Translation, with 6 consistent codings and 3 inconsistencies (66.67% agreement). Overall, across all five coding categories, the agreement rate was 79.02% (162 consistent codings, 43 inconsistencies out of 205 total codings).

The result of Cohen’s Kappa as a chance-corrected measure of rater agreement was Cohen’s Kappa = 0.74, which falls within the “substantial” agreement range [36] (Table 1). Nonconformities were resolved by consensus, adding additional categories as necessary and supplementing previous categories and definitions, coding notes, and cues.

**Table 1:** Cohen’s kappa as a chance-corrected measure of rater agreement

		Person 1		
		1	0	
Person 2	1	a = 162	b = 28	190
	0	c = 15	0	15
		177	28	205

*P(observed) = Po = a/(a + b + c) = 0.79; P(chance) = Pc = 1/Number of codes = 1/5 = 0.20; **Kappa = (Po– Pc)/1– Pc) = 0.74**. If there is an unequal number of codes per segment or if only one code is to be evaluated: P(chance) = Pc = Number of codes/(Number of codes + 1)^2^ = 0.14; **Kappa = (Po– Pc)/(1– Pc) = 0.76**

## Results

For each research question, frequency analyses were conducted based on the assigned codes [28]. The distribution of codes showed that “Innovative Individualized Treatments” had the highest mention rate at 20%, particularly in discussions about global research trends (Q1). “Prevention, Recovery, and Participation” was referenced throughout, with frequencies ranging between 10% and 15%, while “Translation” was most prevalent in Q3 (14%), highlighting its relevance in bridging the gap between research and clinical practice. The most frequently mentioned words included Health (*n* = 81, 1.74%), People (*n* = 66, 1.42%), Mental (*n* = 64, 1.37%), Care (*n* = 35, 0.75%), Treatment (*n* = 35, 0.75%), and Research (*n* = 32, 0.69%). Other relevant terms such as Clinical, Outcomes, Interventions, and Disorders appeared with lower but notable frequencies, ranging from 0.3 to 0.5% of the transcript.

### Research trends in mental health

The first discussion question focused on international research trends, with participants identifying several key developments shaping the field. A central challenge discussed was the limited understanding of the brain’s role in mental health, making effective treatment difficult. Experts pointed to major neuroscience initiatives, such as the U.S. Brain Initiative, which aims to improve brain-mapping technologies and expand research on neurobiological mechanisms of mental disorders.

A significant topic was the Research Domain Criteria (RDoC) framework, which moves away from traditional diagnostic categories and instead focuses on transdiagnostic dimensions of mental health conditions [37]. In the field of global mental health, there has been an increasing emphasis on mental health promotion and the development of evidence-based strategies tailored to different sociocultural contexts [38]. Experts emphasized that RDoC’s mechanism-based approach could improve diagnostic accuracy and treatment targeting.

Another emerging trend discussed was phenotyping and precision psychiatry, particularly through digital health tools and computational models. The integration of biological, clinical, and omics data allows for a more individualized approach to mental health care. One participant underscored the role of Computational Psychiatry in bridging gaps in brain function research:

*“We can’t really correct the brain*,* but we may be able to translate it across species and study smaller organisms to gain insights into larger ones. Computational psychiatry allows us to model the brain like a computer*,* making cross-species comparisons possible.”*

Another key research focus was substance use and psychiatric comorbidity, particularly in South Africa, where research explores the interplay between mental disorders and addiction. This topic also sparked broader discussion about global disparities in care, as experts emphasized that access to adequate treatment for substance use disorders remains highly unequal across regions. Under-resourced health systems were seen as facing disproportionate challenges due to limited services and a high burden of psychiatric comorbidity [39]. Ongoing debates on cannabis-induced psychosis and the need for more longitudinal studies to clarify causality were also discussed.

The role of social determinants of mental health was likewise emphasized. Participants agreed that poverty, social exclusion, stigma, and extreme adverse experiences (e.g., violence, incarceration, COVID-19-related losses) are increasingly being recognized in high-impact publications. One expert noted:

*“Mental health research must focus on the impact of violence within families and communities*,* gender discrimination*,* and the marginalization of refugee and immigrant populations.”*

Finally, experts highlighted early intervention as a key strategy to reduce disability and prevent chronicity. The DZPG could play a leading role in coordinating standardized early interventions for serious mental disorders. They stressed that individuals with lived experience should be actively involved in research to ensure interventions are accessible and destigmatized, particularly for children and adolescents.

### Gaps and needs in clinical practice

The second question explored unmet needs in mental health care, with a focus on comorbidity, treatment accessibility, and intervention effectiveness. A major theme was the high mortality rates among people with mental disorders, often due to co-occurring physical conditions. One expert stressed:

*“Treating depression improves outcomes for patients with heart disease and stroke*,* yet we still struggle to reach and treat these populations effectively.”*

Experts also highlighted inequities in mental health service provision, particularly in resource-limited settings. The divide between public and private healthcare was seen as problematic, with the public sector often lacking resources while private-sector patients risk overtreatment.

Treatment resistance was another key issue, emphasizing the need for multimodal interventions combining pharmacotherapy, psychotherapy, and neuromodulation. However, such approaches remain underutilized due to a lack of clinical implementation research.

### Addressing translational gaps in clinical practice

Participants identified clinical outcome monitoring as a major translational gap. Current systems prioritize documentation over treatment effectiveness, leading to a disconnect between clinical interventions and real-world outcomes [40]. Large-scale implementation programs, such as the UK’s Improving Access to Psychological Therapies (IAPT) initiative, have demonstrated how structured, evidence-based psychological treatments can be successfully scaled up and evaluated in routine care [41]. However, challenges remain in ensuring sustainable funding, high-quality therapist training, and consistent treatment fidelity across different settings. One expert illustrated this with an example from the U.S.:

*“We have countless mandatory reports in mental healthcare*,* yet no one measures whether treatments are actually improving patients’ lives. A learning healthcare system must prioritize outcome monitoring.”*

Another issue raised was the exclusion of high-risk populations in research, which limits the generalizability of clinical findings. One expert noted:

*“We lack biological data on the populations most affected by mental disorders*,* which limits the generalizability of clinical biomarkers.”*

Experts called for more inclusive research and the development of clinical guidelines based on real-world effectiveness, rather than controlled trial outcomes alone.

### Opportunities and challenges for the German center for mental health

The final discussion focused on opportunities and challenges within the research domains:

*“High somatic comorbidity and increased mortality”*

Experts emphasized the need to integrate mental health screenings into routine care for chronic illnesses, ensuring early detection and treatment of comorbid conditions.

*“Impact of poverty, social exclusion, stigma, and discrimination”*

Participants viewed this as a complex but essential challenge requiring collaboration across disciplines. Research has demonstrated that social disadvantage is not only linked to an increased prevalence of mental health conditions but also to a reduced likelihood of receiving adequate mental health care [40, 41]. This highlights the need for targeted interventions that address both individual and structural barriers to care. One expert highlighted:

*“Many countries allocate insufficient mental health resources*,* disproportionately excluding the poor. We need strong advocacy efforts to influence policy and funding decisions.”*

The German Center for Mental Health’s focus on marginalized groups was praised as an important step toward improving service accessibility. Experts also cautioned against simplistic assumptions that poverty directly causes mental illness, stressing that access to high-quality care is the more critical factor.

*“Leveraging behavioral, computational, social, and neurobiological sciences for better treatments”*

Computational approaches were identified as a major opportunity, particularly in AI-driven treatment matching. However, experts warned that poorly curated data could compromise predictive modeling. One participant noted:

*“AI has immense potential*,* but reliable outcome monitoring must first be established to create high-quality datasets.”*

Overall, the focus group discussion successfully identified key research trends, clinical challenges, and translational barriers relevant to the German Center for Mental Health. Experts agreed on the need to integrate neuroscience, social determinants, and computational methods to drive innovation in mental health research and clinical care [40].

## Discussion

### Summary

This study provides an essential foundation for the establishment of the German Center for Mental Health (DZPG) by identifying key international research trends, clinical gaps, translational challenges, and research opportunities. The findings highlight critical aspects that will shape the center’s future research agenda.

Regarding global research trends (Q1), discussions emphasized the need for a better understanding of the brain and its link to neuroscience in order to address the high mortality and comorbidity of mental and somatic disorders including addiction. The Research Domain Criteria (RDoC) framework, which moves beyond traditional diagnostic categories, was seen as a promising approach for the DZPG. This study aligns with the “Grand Challenges” outlined by Collins & Patel [42], which emphasize the need for interdisciplinary and global collaborations in mental health research. Addressing social determinants and scaling up evidence-based interventions in diverse sociocultural settings remain key challenges for the field. The importance of phenotyping in precision psychiatry was underlined as an essential but underdeveloped area. Experts agreed that social inequalities, urban mental health, and climate change require stronger research focus, all of which will be central elements of the DZPG’s main research domains. Additionally, coordinated and standardized early intervention was recognized as a key priority for the DZPG, ensuring timely prevention and treatment of mental health disorders. Early intervention is a cornerstone of modern psychiatric research, as emerging evidence suggests that proactive treatment approaches can prevent chronicity and long-term disability [43–45]. The DZPG’s research agenda integrates these insights by prioritizing innovative strategies for the early detection and management of mental disorders.

For gaps in clinical practice (Q2), the discussion centered on the high premature mortality of people with mental disorders, particularly due to comorbid physical conditions. Participants emphasized the importance of understanding why some interventions succeed while others fail and the need for more personalized treatment strategies. The DZPG will focus on improving treatment-resistant symptoms and ensuring equitable healthcare access, particularly for vulnerable populations.

Addressing translational gaps (Q3), experts stressed the lack of real-world outcome data on treatment effectiveness and the urgent need for a learning healthcare system that prioritizes patient-centered care. Translation efforts must extend beyond controlled clinical settings to high-risk populations, ensuring gender, ethnic, and socioeconomic diversity in research and practice.

In the final discussion on opportunities and challenges for the DZPG (Q4), participants highlighted the integration of mental health screenings into routine care, particularly in managing chronic illnesses and multimorbid conditions. Social exclusion, stigma, and poverty were identified as major barriers to care, requiring collaboration with policymakers and community stakeholders. The role of social determinants in mental health cannot be overstated. Research has shown that individuals in socioeconomically disadvantaged areas are at significantly higher risk for psychological distress, often due to structural inequalities and limited access to care [46]. Computational approaches and AI-driven models were recognized as promising tools for identifying comorbidities and risk patterns, provided they are backed by high-quality, diverse datasets.

Another key implication of this study is the need to strengthen education and public awareness in mental health. Enhancing mental health literacy among clinicians, patients, families, and the public is essential to reducing stigma and improving early intervention [42, 47]. Training programs should integrate knowledge on social determinants of health and community-based interventions to ensure broader societal impact [48]. Evidence suggests that a lack of mental health literacy remains one of the primary barriers to help-seeking, particularly among young people [49]. Public education campaigns have shown effectiveness in reducing stigma and increasing willingness to seek professional support [50].

As a leading research institution, the DZPG is well-positioned to embed educational initiatives into its translational efforts, fostering interdisciplinary collaboration to bridge the gap between research and practice.

These findings are directly relevant to the establishment of the DZPG, aligning with its three research domains. Future research should integrate participatory and interdisciplinary approaches to optimize mental health care and policy implementation. The insights gained from this study have been incorporated into the strategic application for the center’s development and will inform its long-term research and implementation strategies.

### Limitations and further research

Despite the valuable contributions of the experts, this study has several limitations. While it successfully captured key global research trends in North and Latin America, Europe, Asia, Africa and Australia, only one expert per continent was included. Future studies should diversify the spectrum of experts and include experts by experience.

The nature of the discussion was less controversial than expected. This may be attributed to the scientific excellence of the expert panel, as most participants had previously shared their views, data and interpretations in multiple high ranking international conferences. Future studies should integrate perspectives from additional stakeholders, including patients, families, therapists, startups, and industry representatives, to ensure a more diverse range of viewpoints.

The virtual format, necessary due to the COVID-19 pandemic, also presented challenges. While the focus group method provided valuable expert insights, its interactive nature may have led to a convergence of opinions, limiting the diversity of perspectives. An alternative approach, such as the Delphi technique, could be considered for future studies, allowing for iterative expert consensus building in a more controlled, anonymous format [51, 52].

Another limitation was that participants reported difficulties due to time zone differences, with some joining very early in the morning or late at night. While an in-person meeting would have required more logistical effort, it might have enhanced engagement, discussion dynamics, and networking opportunities.

Furthermore, while more participants were initially invited, several were unable to attend due to clinical commitments during the pandemic. However, the smaller group size allowed for in-depth discussions and equal participation.

A final limitation is the lack of prior literature on the newly founded DZPG; thus participants could not be provided in advance with research articles from our center. On the other hand, this underscores the importance of our study, which provides one of the first structured analyses informing the DZPG’s research direction.

### Conclusion

This study serves as a critical framework for the German Center for Mental Health, offering valuable insights into research priorities, clinical needs, and translational strategies. The findings will guide the DZPG in its mission to enhance mental health research, improve patient outcomes, and bridge the gap between science and practice. Given its relevance, this work not only informs the DZPG’s foundation but also sets a precedent for future mental health research efforts in Germany.

## Data Availability

The data that support the findings of this study are available from the first author upon reasonable request.
